# Force-Time Waveform Shape Reveals Countermovement Jump Strategies of Collegiate Athletes

**DOI:** 10.3390/sports8120159

**Published:** 2020-12-02

**Authors:** Trent M. Guess, Aaron D. Gray, Brad W. Willis, Matthew M. Guess, Seth L. Sherman, Dale W. Chapman, J. Bryan Mann

**Affiliations:** 1 Department of Physical Therapy, University of Missouri, Columbia, MO 65211, USA; willisbw@health.missouri.edu (B.W.W.); mmg3nr@mail.missouri.edu (M.M.G.); 2 Department of Orthopaedic Surgery, University of Missouri, Columbia, MO 65211, USA; grayad@health.missouri.edu; 3 Department of Orthopaedic Surgery, Stanford University, Stanford, CA 94305, USA; shermans@stanford.edu; 4 Sport Science, NSW Institute of Sport, Sydney Olympic Park, Sydney, NSW 2127, Australia; Dale.Chapman@nswis.com.au; 5 Department of Kinesiology and Sport Sciences, University of Miami, Coral Gables, FL 33146, USA; bmann@miami.edu

**Keywords:** countermovement jump, force plates, principal component analysis, jump strategy, collegiate athletics

## Abstract

The purpose of this study was to relate the shape of countermovement jump (CMJ) vertical ground reaction force waveforms to discrete parameters and determine if waveform shape could enhance CMJ analysis. Vertical ground reaction forces during CMJs were collected for 394 male and female collegiate athletes competing at the National Collegiate Athletic Association (NCAA) Division 1 and National Association of Intercollegiate Athletics (NAIA) levels. Jump parameters were calculated for each athlete and principal component analysis (PCA) was performed on normalized force-time waveforms consisting of the eccentric braking and concentric phases. A *K*-means clustering of PCA scores placed athletes into three groups based on their waveform shape. The overall average waveforms of all athletes in each cluster produced three distinct vertical ground reaction force waveform patterns. There were significant differences across clusters for all calculated jump parameters. Athletes with a rounded single hump shape jumped highest and quickest. Athletes with a plateau at the transition between the eccentric braking and concentric phase (amortization) followed by a peak in force near the end of the concentric phase had the lowest jump height and slowest jump time. Analysis of force-time waveform shape can identify differences in CMJ strategies in collegiate athletes.

## 1. Introduction

The countermovement jump (CMJ) is a popular assessment of athletic performance with numerous parameters proposed to quantify important aspects of the force–time curves generated during a CMJ. However, debate continues regarding which of these parameters provides the best overall assessment of athletic performance [1,2,3,4,5,6]. Discrete parameters derived from force–time data (e.g., peak force) reduce the dimensionality of the measured waveform [7], facilitating analysis, but important information on how athletes generate force during the jump cycle may be lost. It is invaluable for the practitioner to understand how an athlete applies force and to be able to relate that force application to performance. How the athlete applies force throughout the movement may be of importance for training exercise selection and how to further enhance athletic performance. For example, explosive jumpers are characterized by the ability to jump high in a short amount of time and can be identified by the reactive strength index modified (RSImod) parameter [8,9,10], which is the ratio of jump height over jump time. However, the RSImod parameter does not provide information on how the athlete developed force throughout the CMJ cycle, information that may help identify the unique jump strategy employed by the athlete.

Movement strategies applied by the athlete are reflected in the ground reaction force-time curve during the CMJ and analysis of force-time waveforms may elucidate information on movement strategies and performance [11]. Greater jump heights are obtained by increasing velocity of the jumper’s center-of-mass at take-off. This velocity is directly related to the vertical impulse force generated between the jumper and the ground and is derived from the area under the force-time curve. The same force impulse can be generated through a lower force applied over a longer time, or higher force applied over a shorter time. The shape of the force-time curve may provide information on how force is developed and utilized during the jump. The final phase of the CMJ prior to take-off, the concentric phase, is most responsible for generating jump momentum at take-off. However, the jump cycle is a continuum, and the concentric phase is strongly influenced by the strategy employed by the athlete prior to entering the concentric phase. The ability to identify efficient and inefficient strategies throughout the jump cycle would allow practitioners to identify specific points in the jump where improvements can be made.

Quantitative analysis of waveforms is simplified through dimensionality reduction techniques that extract waveform features for downstream analyses. Principal component analysis (PCA) has been used to differentiate differences in the kinematic and kinetic waveforms of gait analysis [12,13]. PCA has also been applied to CMJ waveforms to distinguish the effect of training programs [14]. Floria et al. used PCA of force, velocity, and center of mass displacement waveforms to identify differences associated with increased CMJ height following a training intervention for female basketball players [14]. While PCA coefficients can be used in hypothesis testing to determine if differences occurred in waveform shape between existing groups, clustering techniques of PCA coefficients can be used to place athletes in groups based on the similarity of waveform shape [15].

Analysis of force-time waveforms during the CMJ may identify movement strategies employed by athletes and provide a more complete profile of the athlete. This information could aid practitioners in selecting training to enhance athletic performance. Relating movement strategies gleaned from force-time waveforms to sport and performance parameters is a needed step in the realization of this objective. The purpose of this study is to determine differences in jump strategies, using PCA with *K*-means clustering of force plate data, for collegiate athletes at the National Collegiate Athletic Association (NCAA) Division 1 and National Association of Intercollegiate Athletics (NAIA) levels across multiple male and female sports. We hypothesized that linear dimensionality reduction and clustering of force–time waveforms during the CMJ would elucidate differences in jumping strategies for these athletes and that these differences would correlate to gross CMJ parameters. This information could then be used to determine jump strategies of high performing athletes and guide training for sport–specific requirements.

## 2. Materials and Methods

**Figure 1:**
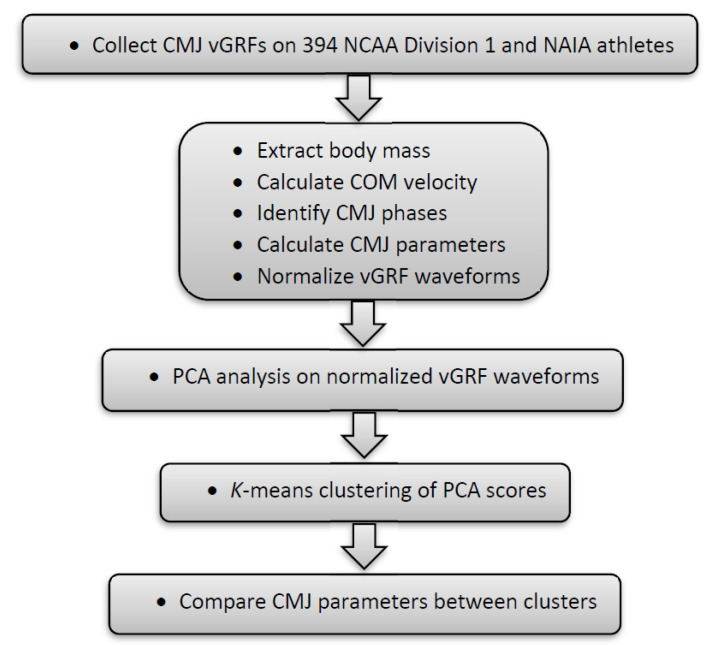
Flow diagram of project. COM is the body center of mass.

Project work (Figure 1) consisted of measuring vertical ground reaction forces (vGRF) for 394 collegiate athletes performing a counter movement jump (CMJ). Discrete CMJ parameters were calculated for each athlete and a principle component analysis (PCA) performed on vGRF waveforms from all athletes. A K-means clustering was used to cluster athletes based on their PCA scores and CMJ parameters were compared between athletes in each cluster.

### 2.1. Participants

Participants were from the midwestern region of the United States and were selected based on participation in a collegiate athletic program. Participants (n = 394, age 18–23 years, mass = 74.0 ± 14.3 kg) included NCAA Division 1 athletes (baseball (n = 38), men’s basketball (n = 12), men’s swimming and diving (n = 26), softball (n = 21), women’s volleyball (n = 7), women’s basketball (n = 11), women’s soccer (n = 20), women’s swimming and diving (n = 20), and wrestling (n = 24)) and NAIA athletes (baseball (n = 15), cheer (n = 9), cross country (n = 17), men’s basketball (n = 13), men’s golf (n = 13), men’s soccer (n = 22), men’s tennis (n = 13), softball (n = 16), track and field (n = 32), women’s volleyball (n = 16), women’s basketball (n = 13), women’s golf (n = 9), women’s soccer (n = 19), and women’s tennis (n = 8)). All subjects gave their informed consent prior to participation. The study was conducted in accordance with the Declaration of Helsinki, and the protocol was approved by our Institutional Ethics Committee (Project # 2009420-QI). The inclusion criteria included participation in collegiate athletics and the exclusion criteria included inability to safely perform a CMJ.

### 2.2. Counter Movement Jump Force Plate Data Collection

Following a standard dynamic warm-up, each participant performed two CMJs without an arm swing [16] with each foot centered on two single-axis force plates (PS-2141, PASCO Scientific, Roseville, CA, USA) sampling at 1000 Hz. The force plates were placed on a solid substrate 9 cm apart within a foam surround, the same height as the force plates, for the purpose of ensuring the safety of the athlete upon landing. Each force plate was calibrated prior to data collection and verified via a calibrated mass. Participants were first instructed to stand motionless, with their arms akimbo, for at least two seconds for the purpose of measuring body mass. Participants were then instructed to jump quickly and as high as possible with their hands remaining on their hips. After landing, they returned to the initial starting position with a foot centered on each plate and remained in a quiet standing position prior to being instructed to jump again.

### 2.3. Body Mass and Center of Mass Velocity

Custom MATLAB code (MATLAB R2019b, The MathWorks Inc., Natick, MA, USA) was used to process the force plate data, identify CMJ phases, and calculate CMJ parameters. Forces from the two force plates were summed to produce total vGRF. A 50 ms window of force data collected during quiet standing was used as an initial estimate of body weight. To ensure the accuracy of body mass measurements, the criteria for establishing body mass required the standard deviation of force during a 1 s window be less than 0.1% of the initial estimate of body weight. Center of mass (COM) velocity was determined from vGRF using the following relationships.

(1) vGRF=m×accCOM+m×g

where vGRF is the total vertical ground reaction force measured by the force plates, m is the body mass of the athlete, and g is acceleration due to gravity. Acceleration of the body’s COM, accCOM, can be determined from Equation (1). Velocity of the COM, velCOM, can be determined by integrating accCOM. To reduce accumulated integration errors over the two jump cycles, velCOM was recalculated for each jump starting at a point 0.2 s prior to the onset of CMJ motion.

### 2.4. Counter Movement Jump Phases and Parameters

**Figure 2:**
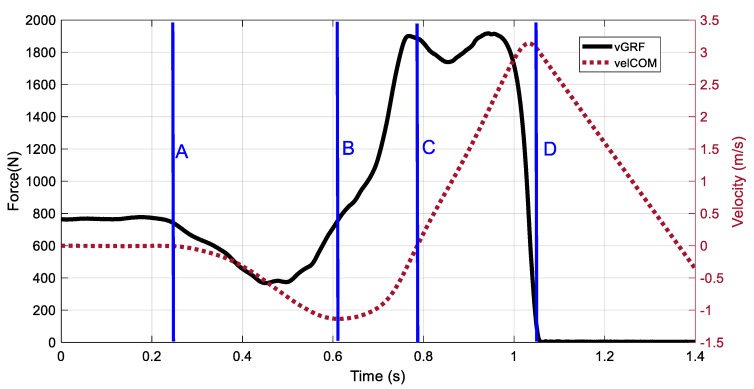
Defined time points of the CMJ. (**A**): onset of motion (vGRF drops below 2.5% body weight), (**B**): start of the eccentric braking phase (peak negative velCOM), (**C**): start of concentric phase (velCOM crosses zero), (**D**): take-off (vGRF drips below 20 N).

Both velCOM and vGRF were used to determine phases of the CMJ cycle (Figure 2). The onset of CMJ motion was defined as the point when the vGRF dropped below 2.5% of body weight following motionless standing [3]. The take-off point of the CMJ was defined as the point when vGRF first dropped below 20 N. Time to take-off was defined as the time from onset of CMJ motion to the time at take-off. The start of the eccentric braking phase [17] was defined as the peak negative velCOM between the onset of CMJ motion and the take-off point. The end of the eccentric braking phase (start of concentric phase) was defined as the point when velCOM crossed zero following the start of the eccentric phase. The eccentric braking and concentric phase times were determined for each CMJ from the time points defining the start of the eccentric braking phase, the start of the concentric phase, and take-off.

Jump parameters calculated for each participant included jump height, eccentric braking phase time, concentric phase time, time to take-off, reactive strength index modified (RSImod), eccentric braking rate of force development (RFD), eccentric braking phase net vertical impulse, concentric phase net vertical impulse, mean eccentric braking power, mean concentric power, peak eccentric braking power and peak concentric power.

Jump height was calculated using velCOM at take-off and changes in kinetic and potential energy between take-off and the point when peak jump height is reached [18]. In this method, jump height is the difference between the height of the COM at the point of take-off and the peak height reached during the jump. The RSImod is a ratio of the jump height over total jump time and has been shown to be a reliable indicator of jump performance [1,9]. RSImod was calculated using jump height divided by time to take-off. The RFD is a measure of how fast the athlete can develop force and was determined using vGRF at the start and end of the eccentric braking phase and the eccentric braking phase time. The RFD was normalized to body mass. The net vertical impulse during the eccentric braking and concentric phases was calculated using the vGRF time integral for each respective phase with body weight excluded. The net force impulse values were normalized to body mass for each subject [6]. The mean and peak powers for each phase were calculated using vGRF and velCOM. Eccentric braking power was reported as a negative value because vGRF and velCOM act in opposite directions. All power values were normalized to body mass. Calculated jump parameters were averaged for each subject over the two measured jumps.

### 2.5. Normalized Ground Reaction Force Waveforms

The analyzed vGRF waveform included both the eccentric braking and concentric phases. The vGRF time series for each jump was truncated to the start of the eccentric braking phase and to the end of the concentric phase (take-off). Each vGRF waveform was normalized in magnitude to body mass and then time normalized by fitting it to a cubic spline that consisted of 101 points. Each participant performed two CMJs and the two normalized waveforms were averaged [19].

### 2.6. Principal Component Analysis and K-Means Clustering

PCA is an orthogonal decomposition technique that reduces large data sets into independent variables representing unique characteristics of the original waveforms called principal components (PCs). PCA reduces the data by extracting PCs that capture the maximum variance in the original waveforms. Typically, only a few PCs are needed to adequately reproduce the original waveform. The PC scores, coefficients that provide the contribution of each PC for an individual subject, are independent and suitable for downstream analyses such as cluster analysis and hypothesis testing of waveform differences. For this study, PCA was conducted on a 394 × 101 matrix (394 participants, 101-point vGRF waveform) in MATLAB. The PCA generated 101 eigenvectors (PC loading vectors) and for each athlete a PC score was generated for every PC loading vector (394 × 101 matrix). The normalized force-time curve for each participant can be reconstructed using Equation (2).

(2) $$\;{\hat{x}}_{i}=\bar{x}+\overset{n}{\underset{j=1}{\sum }}PC\;score_{i}\;\left(j\right)\times \;PC\;loading\;vector\left(j\right)$$

where ${\hat{x}}_{i}$ is the reconstructed normalized vGRF waveform for athlete *i*, *n* is the number of PCs used in the reconstruction, $\bar{x}$ is the mean normalized vGRF, $PC\;score_{i}\;\left(j\right)$ represents the principal component scores for athlete *i*, and PC loading vector(j) represents the loading vectors. If all PCs (*n* = 101) are used in Equation (2), then ${\hat{x}}_{i}$ will be an exact reproduction of the original normalized vGRF waveform. Typically, all components are not needed and only the first few PCs are required to adequately reconstruct the original force–time waveform. Parallel analysis was used to determine the number of PCs to retain [13,20] and based on this analysis, four PCs were retained for our PCA. The first four PCs accounted for 97.6% of the variance in the vGRF waveforms (PC1 = 66.5%, PC2 = 18.5%, PC3 = 8.8%, and PC4 = 3.8%). *K*-means clustering is a method used to partition observations into *k* clusters and when performed on PC scores, *K*-means clustering can identify groups that have similar waveform shapes. *K*-means clustering has previously been found to be suitable for clustering vGRF waveforms [15]. For this study, a *K*-means clustering algorithm was run on the four PC scores retained for each athlete using multiple values of *k*. Silhouette analysis was used to evaluate the separation distance between clusters resulting in three clusters being chosen.

### 2.7. Statistical Analysis

*K*-means clustering grouped athletes into three clusters based on their first four PC scores. MATLAB R2019b was used for statistical analysis calculations. Descriptive statistics, including the mean and standard deviation, of body mass, jump height, eccentric braking phase time, concentric phase time, time to take-off, RSImod, eccentric braking RFD, eccentric braking impulse force, concentric impulse force, mean eccentric braking power, mean concentric power, peak eccentric braking power, and peak concentric power were calculated for athletes in each of the three clusters. Descriptive statistics of PC scores for the four PCs retained were also calculated for each cluster. In addition, the mean and standard deviation of the normalized vGRF waveforms for each cluster were calculated. To examine the differences in means for each cluster, the 95% confidence interval of body mass, CMJ parameters, and PC scores for athletes in each cluster were calculated. Alternatively, a single factor ANOVA was used to determine if there were statistically significant differences (statistical significance level set at *p* ≤ 0.05) across cluster means for body mass, CMJ parameters, and PC scores. Where statistically significant differences occurred, Tukey’s Honest Significant Difference (HSD) post hoc analysis was performed to detect differences between individual clusters. In addition, the magnitude of Cohen’s *d* effect size between individual clusters was calculated for all CMJ parameters and PC scores that had statistically significant differences across clusters (PC1, PC2, and PC3). To determine the distribution of athletes in each cluster by sport, the frequency of athletes in each cluster was recorded for each team in a contingency table. A Pearson’s chi-squared test for independence was used to determine if the team cluster distributions occurred by chance or if there was a statistically significant difference between observed distribution and an expected distribution of no relationship between cluster and sport. Pearson residuals were used to determine the extent of the difference between the expected and actual cluster distribution for each team. Pearson residuals greater than 3 indicate that a team had a high concentration of athletes in that cluster.

## 3. Results

**Figure 3:**
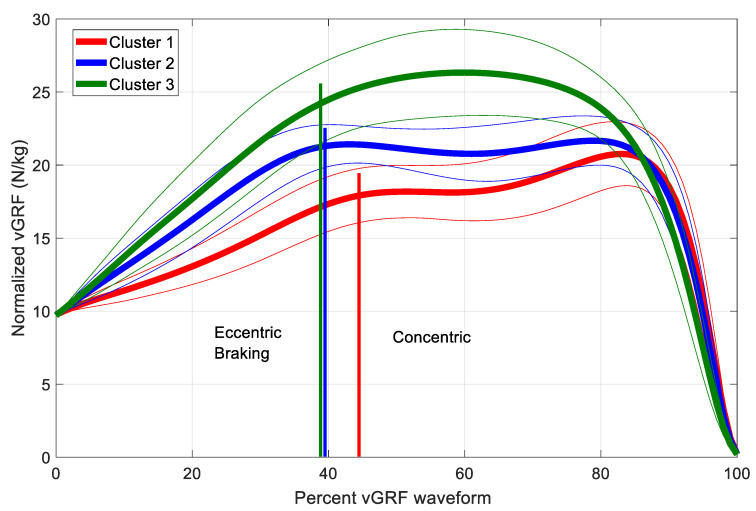
Average vGRF waveforms (eccentric braking and concentric phases) for each cluster. The average transition point, from eccentric braking to the concentric phase, is also shown for each cluster as a vertical line.

*K*-means clustering of the vGRF waveform PC scores grouped 171 athletes into Cluster 1, 165 athletes into Cluster 2, and 58 athletes into Cluster 3. The average vGRF waveforms of athletes in each cluster produced three distinct waveform shapes (Figure 3). Athletes in Cluster 1 generated lower normalized vGRFs throughout the eccentric braking and concentric phases. The shape of the Cluster 1 average waveform is characterized by a plateau at the start of the concentric phase and then a peak just prior to the rapid decrease in vGRF at the end of the concentric phase. The shape of the Cluster 2 average waveform is characterized by two peaks of approximately equal magnitude, one occurring at the start of the concentric phase and the second peak occurring at just prior to the rapid decrease in vGRF at the end of the concentric phase. Athletes in Cluster 3 generated the greatest vGRF, with the waveform shape being a single hump peaking approximately at 1/3 of the way through the time in the concentric cycle. The Cluster 3 peak occurred at roughly the same time as the trough between concentric peaks for Cluster 2 athletes and at the end of the concentric plateau for Cluster 1 athletes.

Athletes in Cluster 3 jumped significantly higher than athletes in Cluster 1 and on average jumped higher than athletes in Cluster 2, although this difference was not significant. Athletes in Cluster 3 jumped in less time than athletes in Clusters 1 and 2 (Table 1), with shorter time to take-off and less time spent in both the eccentric braking and concentric phases. Athletes in Cluster 1 had the lowest jump heights and longest jump times. Cluster 3 athletes had higher RSImod and higher eccentric braking RFD than Cluster 1 and 2 athletes, with Cluster 1 athletes having the lowest values. Cluster 2 athletes generated the greatest net vertical force impulse during the eccentric braking phase, with Cluster 1 athletes generating the lowest force impulse. Cluster 3 athletes generated the greatest net vertical force impulse during the concentric phase, with Cluster 1 athletes generating the lowest. Cluster 2 and 3 athletes generated similar mean and peak power during eccentric braking, with Cluster 1 athletes producing lower power. Cluster 3 athletes generated the greatest mean and peak power during the concentric phase and Cluster 1 athletes generated the lowest power.

Differences in body mass across the three clusters were not statistically significant (Table 1). There were statistically significant differences across clusters for every evaluated jump parameter. Post hoc analysis showed statistically significant differences between groups with the exception of jump height, net concentric impulse, mean eccentric braking power and peak eccentric braking power between Clusters 2 and 3. All differences in jump parameters between Clusters 1 and 2 and between Clusters 1 and 3 were statistically significant. In addition, Cohen’s *d* effect size between Cluster 1 and Cluster 3 was large (>0.8) for all measured jump parameters. The largest effects sizes occurred for eccentric braking RFD, where effect sizes where greater than 2.1 for all cluster comparisons and for eccentric braking time where all effect sizes were greater than 1.6 for all cluster comparisons.

**Figure 4:**
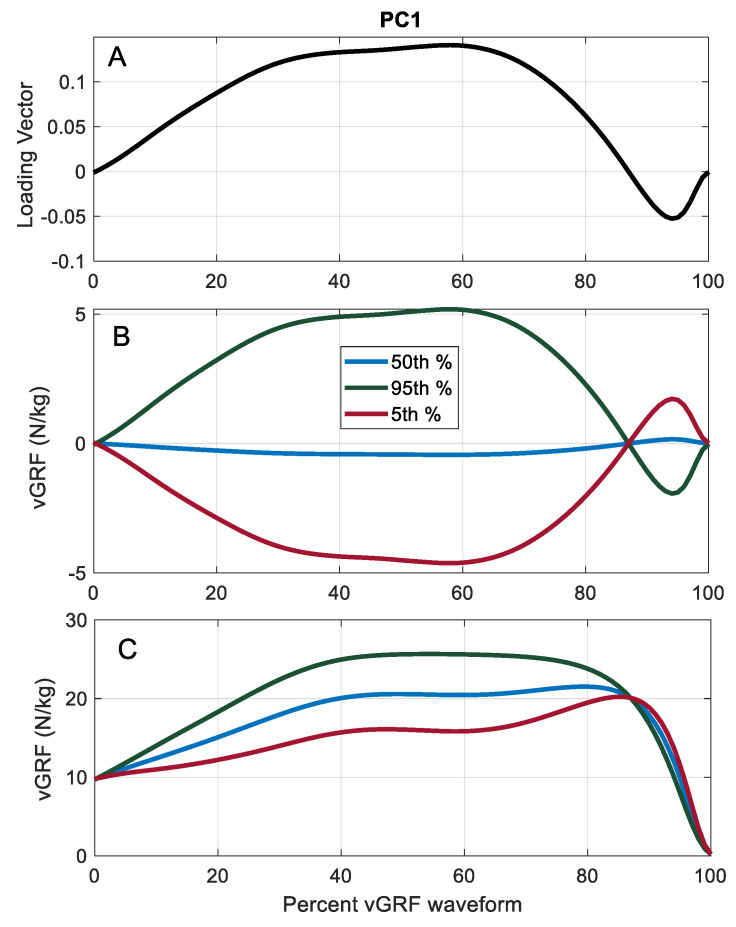
Loading vector for PC1 (**A**); PC1 loading vector multiplied by 5th, 50th, and 95th% PC1 scores (**B**); reconstructed vGRF waveforms using only PC1 with 5th, 50th, and 45th% PC1 scores (**C**).

There were statistically significant differences across clusters for PC scores 1, 2, and 3 (Table 2). PC score differences between clusters were significant with the exception of PC2 between Clusters 1 and 3, and PC3 between Clusters 1 and 2 and between Clusters 1 and 3. PC1 explains 66.5% of the vGRF waveform variance and PC1 scores are significantly different across and between the three clusters. In addition, the Cohen’s *d* effect sizes between clusters for PC1 were extremely large with all values greater than 2.6. To better understand the contribution of PC1 to the vGRF waveform shape, the waveform was reconstructed (Equation (2)) using only PC1. Three waveforms were reconstructed using PC1 scores at the 5th, 50th, and 95th percentile of PC1 (Figure 4). The resulting waveform shapes are similar to the averaged waveforms of Clusters 1, 2, and 3 (Figure 3).

There was a statistically significant relationship between sport and cluster frequency (Pearson’s chi-squared test *p*-value < 0.01). Sports that had a high distribution of athletes in Cluster 1 were NAIA cross country, D1 women’s soccer, NAIA softball, and NAIA women’s golf (Table 3). Sports with a high distribution of athletes in Cluster 2 were D1 men’s swimming, NAIA men’s basketball, and NAIA men’s tennis. Sport with a high distribution of athletes in Cluster 3 were D1 men’s basketball, D1 wrestling, D1 women’s basketball, and D1 men’s swimming.

## 4. Discussion

The purpose of this study was to relate the shape of CMJ vGRF waveforms to discrete jump parameters and determine if waveform shape could enhance CMJ analysis. In this study, collegiate athletes were clustered into three groups based on the shape of their vertical ground reaction force–time waveform recorded with a force plate during akimbo counter-movement jumping. Specifically, *K*-means clustering was performed on PC scores resulting from PCA of vGRF waveforms consisting of the eccentric braking and concentric phases of CMJs. There were statistically significant differences in every calculated jump parameter across the three vGRF shape-based clusters. Athletes clustered into Cluster 3 were high performing athletes characterized by high jump heights, short jump durations, high rate of force development and high jump power. Athletes in Cluster 1 were lower performing jumpers with lower jump heights and longer jump duration.

The transition from eccentric braking to the concentric phase occurs at the point of lowest displacement of body COM. The small region around this transition is often referred to as the amortization phase of the CMJ [3]. Cluster 1 athletes have a plateau in vGRF following amortization, while Cluster 2 athletes have a peak in vGRF at amortization. Cluster 3 athletes continue developing force through the amortization phase with a peak in vGRF towards the middle of the concentric phase (Figure 3). The vGRF shape of Cluster 3 athletes may indicate better utilization of the muscle tendon unit stretch-shortening cycle and a more efficient jump strategy [21]. Cluster 3 athletes exhibit greater RFD which has been associated with muscle tendon properties [22]. RFD during the eccentric braking phase will also influence concentric phase performance [23]. Cluster 3 athletes enter the concentric phase with higher vGRF which allows them to produce net vertical force impulse at a higher rate and shorter amount of time. Force impulse is directly related to a change in velocity through the impulse–momentum relationship with a higher force impulse resulting in increased velCOM and greater jump height.

The eccentric braking and concentric phases of the CMJ produce positive net impulse and the waveforms during these phases were chosen for analysis. The unloading and eccentric yielding phases (also collectively called the unweighting phase [17,23]) of the CMJ were not included in the vGRF waveforms of this study. The unloading and eccentric yielding phases include onset of motion (point A, Figure 2) to the start of eccentric braking and can provide information on how athletes enter the eccentric braking phase [23]. Although not explicitly included in our PCA, the CMJ strategy of the three clusters during the unloading and eccentric yielding phases (unweighting phase) can be inferred from the eccentric braking and concentric vGRF waveform shapes. The slope of vGRF, or rate of force development, is greatest for the Cluster 3 athletes and smallest for Cluster 1 athletes. This would infer that in order to achieve the higher slope in vGRF throughout eccentric braking, Cluster 3 athletes achieved greater reductions in vGRF during unweighting with an associated increase in COM displacement (towards the ground) and increase in peak negative velCOM. Future studies would benefit from including the entire CMJ cycle in PCA waveforms as unweighting influences vGRF behavior in the eccentric braking phase which in turn influences vGRF and jump performance in the concentric phase.

Cluster 3 athletes produced similar (differences not statistically significant) jump heights as Cluster 2 athletes, but did so with reduced time to take-off and reduced time in the eccentric braking and concentric phases (Table 3). Cluster 2 and 3 athletes have similar net vGRF impulse during the concentric phase, but Cluster 2 athletes have greater eccentric braking net vGRF impulse. Cluster 2 and 3 athletes generate similar power during eccentric braking, but Cluster 3 athletes produce more power during the concentric phase. Cluster 3 athletes had greater RSImod values as well as greater eccentric braking RFD. Although the jump heights are similar, the Cluster 3 vGRF waveforms result in more explosive, quicker jumpers.

Cluster 1 athletes have a vGRF waveform shape associated with the lowest jump heights and the longest jump times. While the vGRF waveform shape of Cluster 3 athletes is characterized by a single hump during the concentric phase, Cluster 1 athletes are characterized by a plateau in vGRF at the start of the concentric phase followed by an increase in vGRF that peaks just before the rapid decrease in vGRF prior to take-off. The Cluster 1 vGRF waveform results in an inefficient CMJ. PC1 explains 66.5% of the variance in vGRF waveform shape and PC1 scores are significantly different between Cluster 1 and Cluster 3. The shape of the PC1 loading vector is characterized by a large hump that peaks at around 55% of the way through the vGRF waveform, consisting of the eccentric braking and concentric phases. PC1 also has a peak in the opposite direction at around 95% of the way through this waveform. A high positive PC1 score produces a waveform similar to the higher performing Cluster 3 athletes and a high negative PC1 score produces a waveform similar to the lower performing Cluster 1 athletes. Qualitative inspection of the CMJ waveform shape could determine whether an athlete is an explosive jumper or a worse performing jumper. Compared to discrete CMJ parameters, analysis of the force–time curve may provide practitioners with a more complete picture of how the athlete generates force during ballistic movement. The shape of the vGRF waveform during CMJs reveals the jump strategy used by individual athletes, information that the practitioner could use to tailor training exercises to enhance desired performance.

Two distinct force-time waveform shapes, consisting of either one peak (unimodal) or two peaks (bimodal), have been observed during the CMJ [24,25,26]. In their analysis of force–time waveforms for 33 athletes, Kennedy and Drake [26] concluded that a bimodal force-time curve was not an optimal shape for jump performance. In a similar study on 100 athletes, McHugh et al. [25] determined that a peak force occurring at the low position of the COM (amortization phase) was a better indicator of jump performance, as determined by jump height and reactive strength index, than whether the waveform shape was unimodal or bimodal. The findings of the current study are generally in agreement with the conclusions of both Kennedy and Drake and McHugh et al., but may offer more insight into the relationship between waveform shape and jump performance. In the current study, the lowest performing jumpers, Cluster 1 athletes, had a bimodal shape with the second peak larger than the first peak. Cluster 2 athletes where characterized by a bimodal shape, with a large first peak occurring at amortization. The highest performing jumpers, Cluster 3 athletes, had a unimodal waveform with a peak that, on average, occurred after amortization (Figure 3).

Cluster 3 athletes were concentrated in the D1 sports of men’s and women’s basketball, men’s swimming, and wrestling. These are highly competitive sports where athletes would benefit from explosive movement. The sports of D1 women’s soccer, NAIA cross country, NAIA softball, and NAIA women’s golf had the highest concentrations of Cluster 1 athletes and are sports where explosive movement may not be as important. The sports of D1 men’s swimming, NAIA men’s basketball, and NAIA men’s tennis had high concentrations of Cluster 2 athletes. The cluster distribution of D1 and NAIA men’s basketball highlights differences in athletes at different competition levels. NAIA men’s basketball athletes were predominately in Cluster 2, while D1 basketball players were predominately in Cluster 3, indicating that while jump heights were similar, D1 athletes were more explosive jumpers.

Due to time constraints of the athletes, we were limited to two CMJs per athlete. Using three or more CMJ trials for analysis is common [3,11], and two trials may not be sufficient to capture intrasubject variability. To address this, our PCA was repeated using only the first CMJ for each athlete. All athletes were placed in the same cluster regardless of whether one or two jumps were used. The sport for each measured athlete was known and this study includes the distribution of athletes in each cluster by sport. A limitation of the work is that cluster distributions for different positions within each team (e.g., distance swimmer versus sprint swimmer) were not determined. Sex was also not considered in this study. Previous studies have found significant differences in jump height between males and females [27], as well as sex differences in jump strategy [28].

## 5. Conclusions

*K*-means clustering of PCA scores of CMJ vGRF waveforms placed athletes into clusters characterized by distinct force–time waveform patterns. There were significant differences in all twelve calculated jump parameters across clusters. Athletes with a unimodal shape were the highest performing jumpers with respect to jump height and jump time. Athletes with a bimodal shape characterized by a larger second peak were the poorest performing jumpers with respect to jump height and jump time. Analysis of force–time waveform shape provides insight into CMJ strategies of collegiate athletes that maximize jump performance. Additionally, waveform shape can provide practitioners with a qualitative means for CMJ evaluation.

## Figures and Tables

**Table 1:** The number and percentage of participants in each cluster along with the mean and standard deviations of body mass and CMJ parameters. Also included are the single factor ANOVA *p*-values of body mass and CMJ parameters across all three clusters as well as the Tukey’s HSD post hoc test *p*-values between clusters. Body mass was the only variable that did not show statistically significant differences across the means of the three clusters. * Significant difference (*p* ≤ 0.05).

Parameter	Avg ± Std				Tukey’s HSD		
	95% CI [ ]				Cohen’s *d* Effect Size		
	Cluster 1	Cluster 2	Cluster 3	ANOVA	1 vs. 2	1 vs. 3	2 vs. 3
Number of Participants	171 (43%)	165 (42%)	58 (15%)	n/a	n/a	n/a	n/a
Body Mass (kg)	72.8 ± 15.5 [70.5, 75.1]	74.0 ± 13.2 [72.0, 76.0]	77.3 ± 13.7 [73.8, 80.8]	0.19	n/a	n/a	n/a
Jump Height (m)	0.29 ± 0.09 [0.274, 0.300]	0.34 ± 0.08 [0.324, 0.348]	0.37 ± 0.09 [0.343, 0.390]	<0.01 *	<0.01 * 0.58	<0.01 * 0.89	0.05 0.36
Eccentric Braking Time (s)	0.25 ± 0.05 [0.238, 0.253]	0.18 ± 0.02 [0.177, 0.184]	0.14 ± 0.02 [0.137, 0.148]	<0.01 *	<0.01 * 1.65	<0.01 * 2.31	<0.01 * 1.70
Concentric Time (s)	0.30 ± 0.04 [0.299, 0.310]	0.27 ± 0.03 [0.269, 0.277]	0.22 ± 0.03 [0.212, 0.226]	<0.01 *	<0.01 * 1.00	<0.01 * 2.51	<0.01 * 1.98
Time to Take-Off (s)	0.96 ± 0.11 [0.940, 0.973]	0.82 ± 0.09 [0.811, 0.837]	0.73 ± 0.09 [0.710, 0.755]	<0.01 *	<0.01 * 1.35	<0.01 * 2.15	<0.01 * 1.06
RSImod (ratio)	0.30 ± 0.10 [0.290, 0.319]	0.41 ± 0.09 [0.395, 0.422]	0.51 ± 0.09 [0.472, 0.543]	<0.01 *	<0.01 * 1.13	<0.01 * 1.89	<0.01 * 0.96
Ecc Brak RFD (N/s)/kg	36.66 ± 10.29 [35.1, 38.2]	67.02 ± 13.84 [64.9, 69.1]	109.65 ± 31.17 [101.6, 117.7]	<0.01 *	<0.01 * 2.50	<0.01 * 4.06	<0.01 * 2.15
Net Ecc Brak Impulse (N-s/kg)	0.92 ± 0.17 [0.892, 0.942]	1.13 ± 0.19 [1.105, 1.162]	1.07 ± 0.21 [1.011, 1.121]	<0.01 *	<0.01 * 1.23	<0.01 * 0.83	0.04* 0.35
Net Con Impulse (N-s/kg)	2.33 ± 0.38 [2.28, 2.39]	2.53 ± 0.31 [2.49, 2.58]	2.64 ± 0.34 [2.55, 2.72]	<0.01 *	<0.01 * 0.58	<0.01 * 0.83	0.12 0.33
Mean Ecc Brak Power (W/kg)	−7.81 ± 1.66 [−8.06, −7.56]	−10.77 ± 2.16 [−11.10, −10.44]	−10.81 ± 2.77 [−11.51, −10.09]	<0.01 *	<0.01 * 1.54	<0.01 * 1.50	0.99 0.01
Mean Con Power (W/kg)	22.58 ± 4.76 [21.87, 23.29]	27.01 ± 4.00 [26.40, 27.62]	32.68 ± 5.27 [31.32, 34.03]	<0.01 *	<0.01 * 1.01	<0.01 * 2.06	<0.01 * 1.30
Peak ECC Brak Power (W/kg)	−10.11 ± 2.21 [−10.44, −9.78]	−14.24 ± 3.15 [−14.72, −13.76]	−14.45 ± 4.07 [−15.49, −13.40]	<0.01 *	<0.01 * 1.53	<0.01 * 1.55	0.89 0.06
Peak Con Power (W/kg)	45.22 ± 9.68 [43.76, 46.67]	50.29 ± 8.16 [49.05, 51.54]	57.43 ± 9.61 [54.64, 59.90]	<0.01 *	<0.01 * 0.57	<0.01 * 1.26	<0.01 * 0.83

**Table 2:** The mean and standard deviations of principal component (PC) scores for each cluster along with the single factor ANOVA *p*-values across all three clusters and Tukey’s HSD post hoc test *p*-values between clusters. * Significant difference (*p* ≤ 0.05).

Principal Component	Avg ± Std			ANOVA	Tukey’s HSD		
	95% CI [ ]				Cohen’s *d* Effect Size		
	Cluster 1	Cluster 2	Cluster 3		1 vs. 2	1 vs. 3	2 vs. 3
PC1	−18.87 ± 10.45 [−20.44, −17.31]	6.59 ± 8.46 [5.30, 7.88]	36.90 ± 16.31 [32.70, 41.09]	<0.01 *	<0.01 * 2.67	<0.01 * 4.58	<0.01 * 2.75
PC2	2.63 ± 10.69 [1.02, 4.23]	−4.03 ± 10.85 [−5.69, −2.38]	3.73 ± 13.15 [0.35, 7.12]	<0.01 *	<0.01 * 0.62	0.79 0.10	<0.01 * 0.68
PC3	−0.66 ± 7.01 [−1.71, 0.39]	1.90 ± 7.83 [0.71, 3.10]	−3.47 ± 9.82 [−5.99, −0.94]	<0.01 *	0.01 * 0.35	0.05 0.36	<0.01 * 0.64
PC4	0.46 ± 5.81 [−0.41, 1.33]	−0.59 ± 4.50 [−1.28, 0.10]	0.31 ± 5.67 [−1.15, 1.77]	0.17	n/a	n/a	n/a

**Table 3:** Contingency table of the number of athletes in each cluster by sport with expected values in parentheses. Also shown are the Pearson residuals for each cluster by sport.

	Contingency Table							Pearson Residuals		
	Cluster							Cluster		
Team	1		2		3		Total	1	2	3
D1 Baseball	17	(16.5)	16	(15.9)	5	(5.6)	38	0.51	0.09	−0.59
D1 Men’s Basketball	1	(5.2)	2	(5.0)	9	(1.8)	12	−4.21	−3.03	7.23
D1 Men’s Swimming	0	(11.3)	19	(10.9)	7	(3.8)	26	−11.28	8.11	3.17
D1 Softball	12	(9.1)	7	(8.8)	2	(3.1)	21	2.89	−1.79	−1.09
D1 Women’s Volleyball	2	(3.0)	2	(2.9)	3	(1.0)	7	−1.04	−0.93	1.97
D1 Women’s Basketball	0	(4.8)	6	(4.6)	5	(1.6)	11	−4.77	1.39	3.38
D1 Women’s Soccer	13	(8.7)	6	(8.4)	1	(2.9)	20	4.32	−2.38	−1.94
D1 Women’s Swimming	9	(8.7)	10	(8.4)	1	(2.9)	20	0.32	1.62	−1.94
D1 Wrestling	11	(10.4)	6	(10.1)	7	(3.5)	24	0.58	−4.05	3.47
NAIA Baseball	5	(6.5)	9	(6.3)	1	(2.2)	15	−1.51	2.72	−1.21
NAIA Cheer	6	(3.9)	3	(3.8)	0	(1.3)	9	2.09	−0.77	−1.32
NAIA Cross Country	12	(7.4)	5	(7.1)	0	(2.5)	17	4.62	−2.12	−2.50
NAIA Men’s Basketball	1	(5.6)	10	(5.4)	2	(1.9)	13	−4.64	4.56	0.09
NAIA Men’s Golf	8	(5.6)	4	(5.4)	1	(1.9)	13	2.36	−1.44	−0.91
NAIA Men’s Soccer	10	(9.5)	8	(9.2)	4	(3.2)	22	0.45	−1.21	0.76
NAIA Men’s Tennis	3	(5.6)	9	(5.4)	1	(1.90	13	−2.64	3.56	−0.91
NAIA Softball	11	(6.9)	5	(6.7)	0	(2.4)	16	4.06	−1.70	−2.36
NAIA Track and Field	15	(13.9)	13	(13.4)	4	(4.7)	32	1.11	−0.40	−0.71
NAIA Women’s Volleyball	6	(6.9)	9	(6.7)	1	(2.4)	16	−0.94	2.30	−1.36
NAIA Women’s Basketball	7	(5.6)	5	(5.4)	1	(1.9)	13	1.36	−0.44	−0.91
NAIA Women’s Golf	7	(3.9)	1	(3.8)	1	(1.3)	9	3.09	−2.77	−0.32
NAIA Women’s Soccer	11	(8.2)	7	(8.0)	1	(2.8)	19	2.75	−0.96	−1.80
NAIA Women’s Tennis	4	(3.5)	3	(3.4)	1	(1.2)	8	0.53	−0.35	−0.18
Total	171		165		58		394	-	-	-

Note: Teams with the greatest deviation from expected cluster frequencies are highlighted in green for a high distribution of Cluster 3 athletes, in red for a high distribution of Cluster 1 athletes, and in blue for a high distribution of Cluster 2 athletes.
